# Supplementary material for: Role of the WNT/β-catenin/ZKSCAN3 Pathway in Regulating Chromosomal Instability in Colon Cancer Cell lines and Tissues
Source: Int J Mol Sci. 2022 Aug 18;23(16):9302. doi: 10.3390/ijms23169302 (PMC9409321; doi:10.3390/ijms23169302)
Supplement: Supplementary file 1 [file ijms-23-09302-s001.zip › ijms-1858984-supplementary.pdf]

Supplementary Table S1. Primer sequences used for real-time quantitative PCR.

Gene	Forward primer 5’ to 3’	Reverse primer 5’ to 3’	Size (bp)
PSMD10	GCAGGTTGGTCTCCTCTTCA	GTCTTGGATGTTTGTGGATGCT	294
AURKB	CTGCACCATCCCAACATCCT	GCATCTGCCAACTCCTCCAT	167
ANAPC11	CGGGTAGGGCGAGACGG	CCACAGTTCTCATCGTTGGC	156
PSMB6	GGGAAGGCATGACCAAGGAA	GCGAGAGCTGTGGATAAAAGA	72
CDK1	GGAAGGGGTTCCTAGTACTGC	AGAGTGTTACTACCTTAACAAGTGA	209
BUB3	GCATCGCATCACTTGCCTTC	GGATGATTAGGTGGACTTGGGT	155
MAD2L1	CGTGCTGCGTCGTTACTTTT	ATGGCCAGGGACACAAACAA	109
VPS35	AGAACCCATGTTGTCCTCTGG	AGTGAGCTCTGTGAATATATGAGAA	284
CDC20	AATGTGTGGCCTAGTGCTCC	AGCACACATTCCAGATGCGA	116
ZKSCAN3	GAGCTCTGCCCTTGTCATTC	CTCCCCAGTGTGGATTCTGT	211
CCNC	AGTCGAGCCGAGCTGATTTG	CCTTTTGGCGCTCCTTCAAC	197
CCNT1(a)	AATAGCCCATCCCGTCGTTT	CCAGGGAACTGTGTGAAGGA	179
CCNB2	TGCCTCCCCACTGATAGGAA	AAAGGGCACAATGAAGCACAC	70
BRCA2	CTGAAACTAGGCGGCAGAGG	CAAATCTGTCCCCTCACGCT	115
MAD2L2	TGCATCTCATCCTCTACGTG	TCCTGGATATACTGATTCAGC	118
MCM3	AGTTCGTCCCAAAGTCGTCC	GGGGATTGTTCTCCTCATCCT	143
CDKN2B	GAATGCGCGAGGAGAACAA	CATCATCATGACCTGGATCGC	172
BCL2	TCGCCCTGTGGATGACTGA	CAGAGACAGCCAGGAGAAATCA	134
BCL2L4	TGGCAGCTGACATGTTTTCTGAC	TCACCCAACCACCCTGGTCTT	195
GAPDH	CCACTCCTCCACCTTTGAC	ACCCTGTTGCTGTAGCCA	112

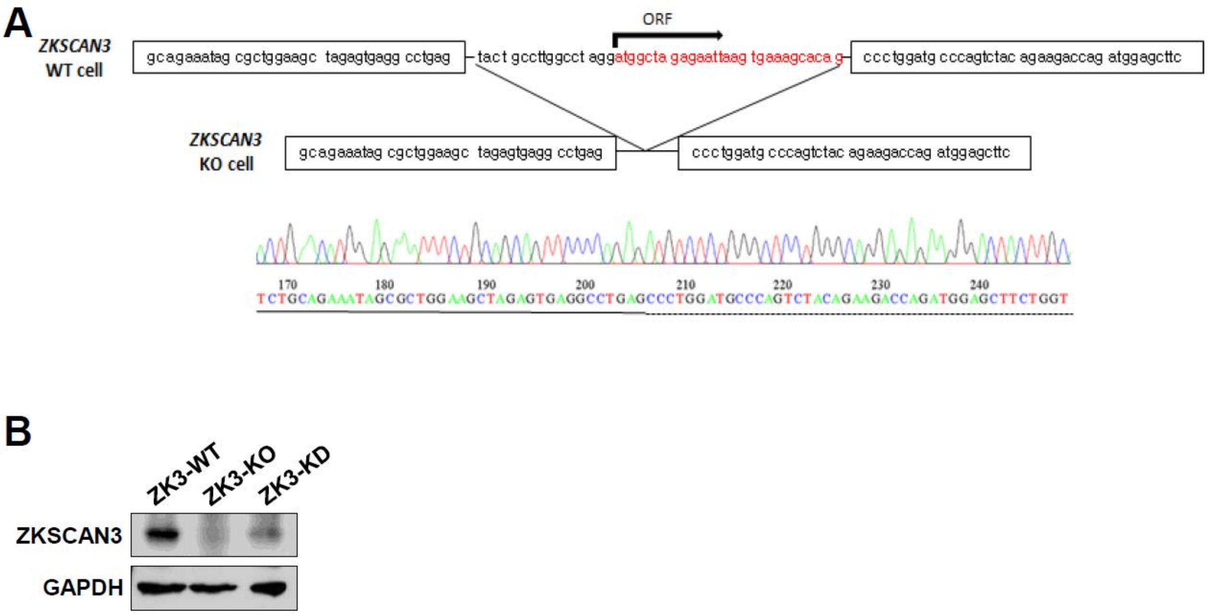

Supplementary Figure S1. (**A**) Construction and sequencing results of ZKSCAN3 knockout cells by CrisPR/Cas9 system are shown. (**B**) Cell extracts were prepared from parental HCT116 (ZK3-WT), homozygous KO (*ZK*-KO), or heterozygous KO (*ZK3*-KD) cells. Cell lysates were then analyzed by western blot for the expression level of ZKSCAN3 using the indicated antibodies.

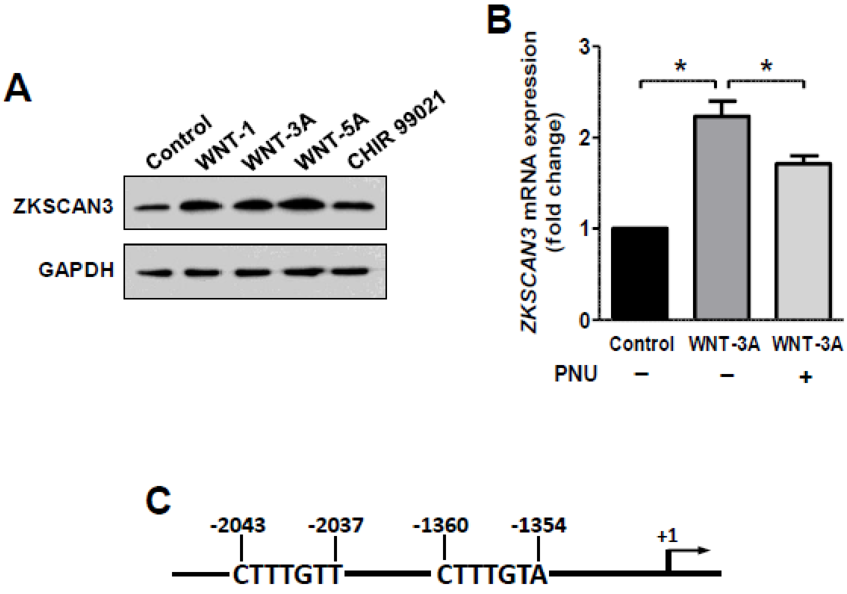

Supplementary Figure S2. (**A**) NCM365D normal colonic epithelial-derived cells were treated with WNT-1 (50 ng/ml). WNT-3A (100 ng/ml), WNT-5A (50 ng/ml), or CHIR 99021 (3 μM) for 24 h, and cell lysates were subjected for western blot using the indicated antibodies. (**B**) Cells were treated with WNT-3A for 24 h in the presence or absence of 40 μ M PNU and then cells were harvested. Extracted mRNA was subjected to qPCR against *ZKSCAN3*. Data represent means ± SD of triplicate measurements. Statistical significance was determined using the Kruskal-Wallis test (* $p < 0.05$). (**C**) Schematic diagram of the human ZKSCAN3 gene promoter.

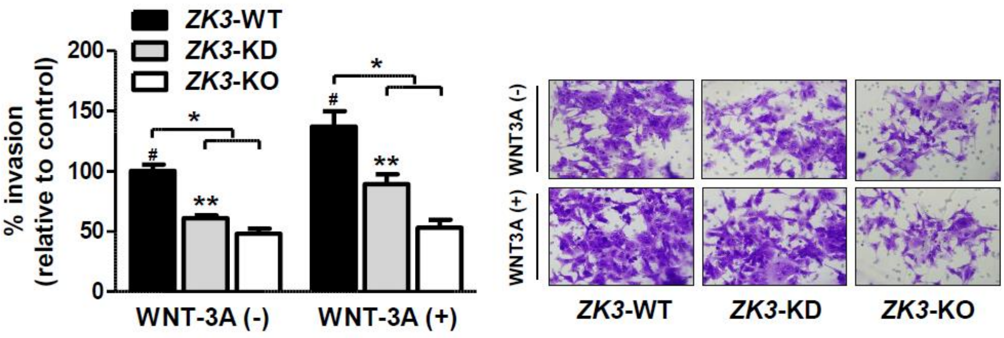

Supplementary Figure S3. Cell invasion assays were performed using a Boyden chamber in the presence or absence of WNT-3A (100 ng/ml) for 24 h. Data shown as percentage of migrated cells relative to control were expressed as means ± SD. Statistical significance was determined using the Kruskal-Wallis test (*, **, # $p < 0.05$). Representative images of the migration assays are presented in the right panel.

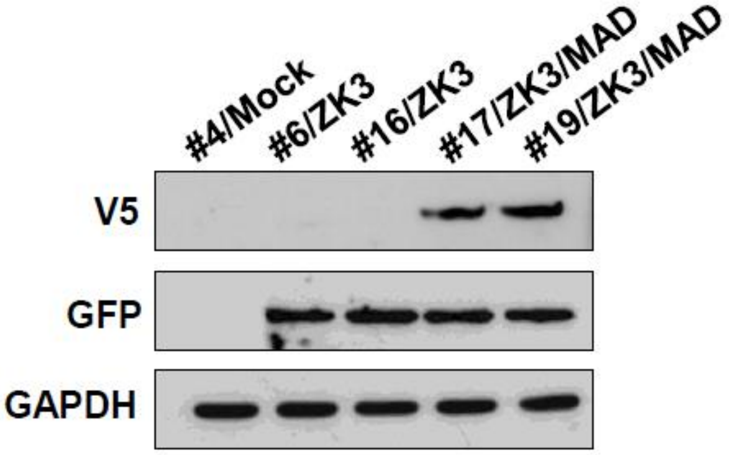

Supplementary Figure S4. Western blot images of HCT116 cells transfected with pcDNAV5 (#4N/Mock), HCT116 cells transfected with pcDNAV5-ZK3 (#6/ZK3 and #16/ZK3), and HCT116 cells co-transfected with pcDNAV5-ZK3 and pDEST-MAD2L2-GFP (#17/ZK3/MAD and #19/ZK3/MAD) using anti-V5, -GFP, and -GAPDH antibodies.

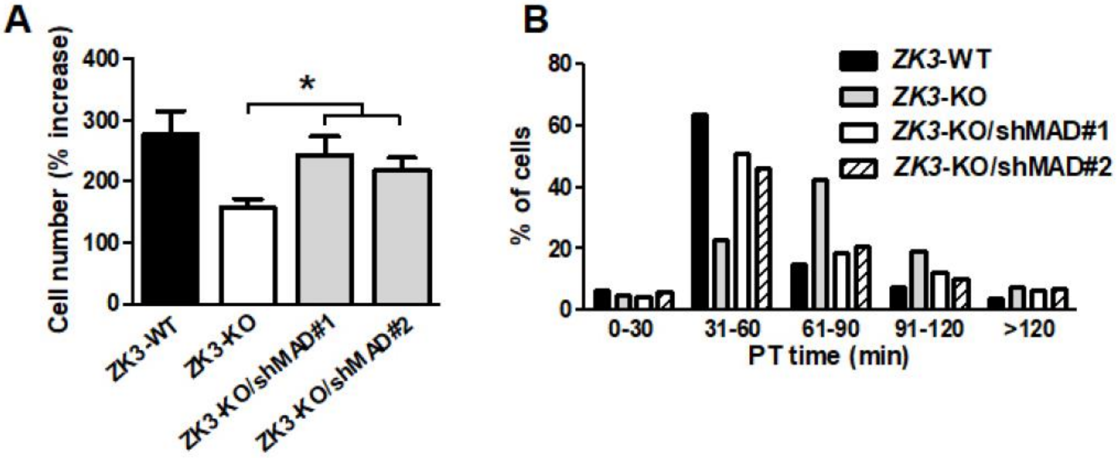

Supplementary Figure S5. (**A**) *ZK3*-KO/shMAD cells were constructed by stably transfecting *ZK3*-KO cells with MAD2L2 shRNA (shMAD). Cell proliferation rate of the indicated cells was determined by sequential monitoring with incuCyte. Presented data obtained 6 days after initial seeding, were expressed as means ± SDs. Statistical significance was determined using the Kruskal-Wallis test (* $p < 0.05$). (**B**) Time-lapse analysis of *ZK3*-WT, *ZK3*-KO, *ZK3*-KO/shMAD#1, and *ZK3*-KO/shMAD#2 cells. Cells stably expressing histone 2B-RFP were cultured in a live cell instrument and PT time was measured. The graph displays the percentage of cells according to time distribution consumed from prometaphase to telophase. At least 50 cells were observed per sample.
